# Barriers and Facilitators to Implementation of Medication Decision Support Systems in Electronic Medical Records: Mixed Methods Approach Based on Structural Equation Modeling and Qualitative Analysis

**DOI:** 10.2196/18758

**Published:** 2020-07-22

**Authors:** Se Young Jung, Hee Hwang, Keehyuck Lee, Ho-Young Lee, Eunhye Kim, Miyoung Kim, In Young Cho

**Affiliations:** 1 Office of eHealth Research and Businesses Seoul National University Bundang Hospital Seongnam Republic of Korea; 2 Department of Family Medicine Seoul National University Bundang Hospital Seongnam Republic of Korea; 3 Department of Pediatrics Seoul National University Bundang Hospital Seongnam Republic of Korea; 4 Department of Nuclear Medicine Seoul National University Bundang Hospital Seongnam Republic of Korea

**Keywords:** clinical decision support system, electronic health record, medication safety, Computerized Provider Order Entry (CPOE)

## Abstract

**Background:**

Adverse drug events (ADEs) resulting from medication error are some of the most common causes of iatrogenic injuries in hospitals. With the appropriate use of medication, ADEs can be prevented and ameliorated. Efforts to reduce medication errors and prevent ADEs have been made by implementing a medication decision support system (MDSS) in electronic health records (EHRs). However, physicians tend to override most MDSS alerts.

**Objective:**

In order to improve MDSS functionality, we must understand what factors users consider essential for the successful implementation of an MDSS into their clinical setting. This study followed the implementation process for an MDSS within a comprehensive EHR system and analyzed the relevant barriers and facilitators.

**Methods:**

A mixed research methodology was adopted. Data from a structured survey and 15 in-depth interviews were integrated. Structural equation modeling was conducted for quantitative analysis of factors related to user adoption of MDSS. Qualitative analysis based on semistructured interviews with physicians was conducted to collect various opinions on MDSS implementation.

**Results:**

Quantitative analysis revealed that physicians’ expectations regarding ease of use and performance improvement are crucial. Qualitative analysis identified four significant barriers to MDSS implementation: alert fatigue, lack of accuracy, poor user interface design, and lack of customizability.

**Conclusions:**

This study revealed barriers and facilitators to the implementation of MDSS. The findings can be applied to upgrade MDSS in the future.

## Introduction

### Background

In 2009, based on evidence that electronic health records (EHR) can improve healthcare quality, the US government enacted the Health Information Technology for Economic and Clinical Health (HITECH) Act [1]. Over the past decade, the healthcare industry has experienced a tremendous digital revolution initiated by the government’s efforts to implement EHRs [2,3]. As of 2017, over 90% of general medical and surgical hospitals in the US use certified EHR systems, thus generating an enormous amount of electronic medical information daily [2,4]. Analysis of big data gathered from EHRs can generate real-time evidence that helps end-users take better care of their patients [5]. Clinical decision support systems (CDSS) are a typical example of value provided to EHR users [6]. Such systems intervene in real-time to help users make appropriate decisions based on up-to-date information from EHRs. Medication decision support systems (MDSS), a well-known and frequently used type of CDSS, reduce adverse drug events (ADE), some of the most common causes of iatrogenic injuries in hospitals [7]. ADEs are generally defined as anticipated or unanticipated side effects resulting primarily from medication errors, attributable to human errors. The most common types of medication errors include the use of contraindicated drugs and overdosing [8]. An MDSS checks for problems based on CDSS data and alerts users in advance of potentially preventable errors. However, despite the high adoption rate of EHRs in the US, ADEs are still a significant problem [9]. The situation is similar in South Korea. ADEs have not been reduced dramatically in South Korea, although the adoption rate of EHRs is around 90% as of 2017 [10,11].

### Prior Research

Efforts have been made to reduce medication errors to minimize the frequency of ADEs. Previous studies have demonstrated that implementing an MDSS in the EHR system improves patient care and overall outcomes by reducing medication errors [12-19]. However, repeated false alerts from MDSSs can decrease healthcare professionals’ productivity by interrupting their workflow [14,20-22]. Furthermore, if doctors are frequently interrupted by false alerts, they are less likely to adopt MDSS recommendations [23].

Research has shown that physicians override about 90% of drug allergy and high-severity drug interaction warning notifications [20,21,24,25]. Two methods could be adopted to improve MDSS. One is to enhance the precision of the MDSS algorithms to reduce unhelpful notifications. Machine learning techniques are being widely considered to provide personalized, accurate notifications [15]. The other method is to support users by understanding the factors associated with MDSS feasibility and usability, which requires an understanding of what factors users consider important in clinical settings. To date, many studies have explored the effectiveness of MDSS, but only a few have analyzed their feasibility and usability.

### Aim

This study aimed to analyze factors related to the adoption of MDSS. A mixed-methods research approach was taken to both quantitatively measure factors necessary for the successful implementation of MDSS and to qualitatively gather and reflect on the opinions of end users. The study encompassed the entire process of implementing an MDSS into a comprehensive EHR system and analyzed the relevant barriers and facilitators. Based on the results, some ideas are suggested to support users and upgrade MDSS, resolving issues already well established by previous studies. 

## Methods

### Design

A mixed research methodology was adopted [26]. Data from a structured survey and 15 in-depth interviews were integrated, and structural equation modeling (SEM) was conducted to yield a quantitative analysis of the factors related to user adoption of MDSS. A qualitative analysis based on semistructured interviews with physicians also collected various opinions about MDSS implementation. To objectively report results, the qualitative analysis followed the Consolidated Criteria for Reporting Qualitative (COREQ) Research Guidelines [27].

### Setting and Participants

The study was conducted at Seoul National University Bundang Hospital (SNUBH), where the comprehensive, privately developed BESTCare electronic medical record (EMR) has been in use since 2003. The system has been accredited three times as a Health Information Management Systems and Society Analytics EMR Adoption Model Stage 7 since 2010. BESTCare implements a proprietary MDSS concurrently with a prescription drug monitoring program run by the South Korean government; thus, BESTCare users are already familiar with MDSS.

A taskforce team of 12 attending physicians, two pharmacists, three nurses, and three engineers was formed to improve medication safety. The team decided to introduce a third-party MDSS to BESTCare. In November 2016, the team analyzed MDSS previously released in the market and decided to implement the Medi-Span solution. The task force analyzed mapping codes, mapping contents, and filters, and designed the user interface and overall system architecture (Table 1).

**Figure 1:**
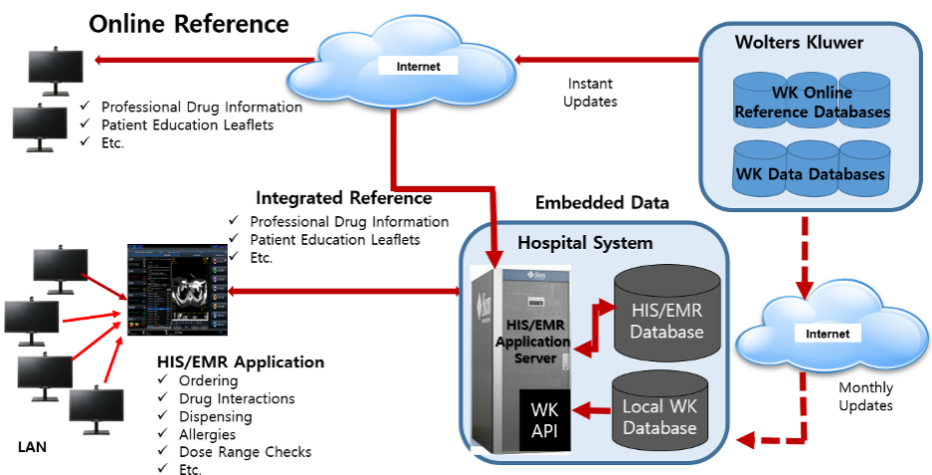
MDSS system architecture and configuration. EMR: electronic medical record; HIS: hospital information system.

**Figure 2:**
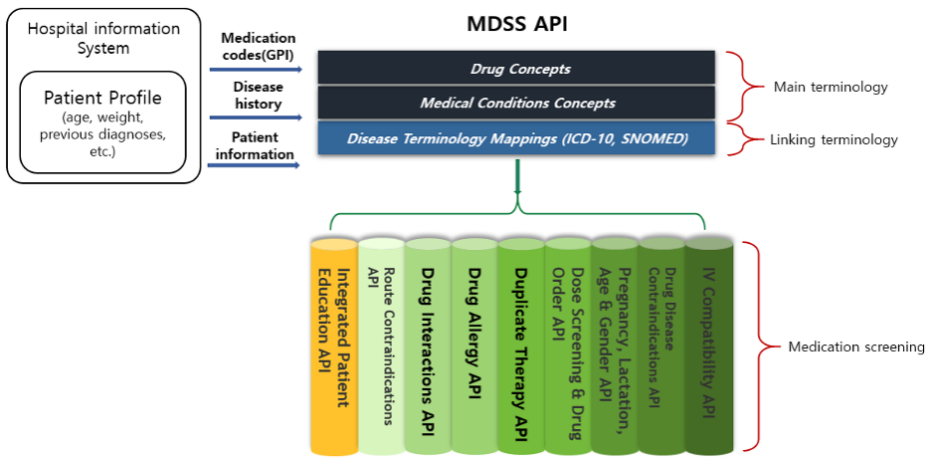
MDSS function list. API: application programming interface; GPI: generic product identifier.

**Figure 3:**
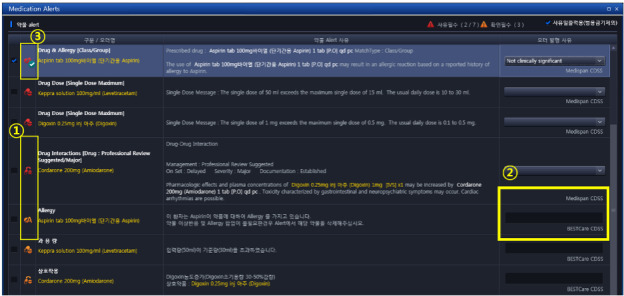
Screenshot of the MDSS user interface. 1) Override requirements: red alerts indicate that users must view an alert message and select a reason for overriding it, whereas orange alerts indicate that users must confirm the alert. 2) Origin of alerts: BESTCare MDSS, Medi-span CDSS, and South Korean national prescription drug monitoring program. 3) Classification of alerts using icons, allowing users to see the notification easily.

The taskforce team designed the overall system architecture of the MDSS (Figures 1 and 2) and designed alert screens to display messages efficiently (Figure 3).

After implementing the MDSS in April 2017, we conducted a structured survey of physicians between May 2017 and October 2017 and employed SEM to analyze the factors facilitating successful implementation. Focused group interviews were also conducted to collect direct opinions from end-users. For the qualitative analysis, study participants were selected through purposive sampling [28], aiming to include participants with in-depth knowledge of the work process involving the EHR system and MDSS. Textbox 1 presents the items included in the semistructured interview questionnaire.

**Table 1:** Basic considerations for integration of a commercialized MDSS into the EMR system.

Mapping	What standard codes should be used to interface Medi-Span with BESTCare?
Contents	What functions should be implemented to improve medication safety? Drug interactions Drug allergies Drug disease contraindications Duplicate therapy Dose screening and drug order Route contraindications Pregnancy/lactation contraindications Gender/age contraindications
Filters	What filters should we integrate? How can we control users’ authorization to override MDSS alerts?
Alerts	How can we show alerts efficiently?
User interface/experience	How can we improve user experience and user interface designs?

**Semistructured interview questionnaire items.**

Questions:

What was your first impression of the MDSS implemented in this hospital?
Did you have experience with other MDSSs before?
How long did it take for you to get used to the MDSS?
Were there any barriers to implementation of the MDSS?
What do you think would help to better implement the MDSS?
How is the navigation when using the MDSS?
Are there any problems with the MDSS that need to be resolved?
Do you think the MDSS is customized well for the EHR workflow? Is there anything missing?
What features or functions do you want to add to the MDSS?
Do you have any recommendations for the MDSS to improve your work experience?
Is there anything else you want to mention regarding the MDSS?

### Data Collection

MYK, a registered nurse, conducted the survey and face-to-face semistructured interviews. IYC, a medical doctor, also led the interviews and took notes. Both interviewers received training on qualitative interviews. The interviews lasted 20 to 60 minutes and were recorded in a closed office or conference room. Nobody was present besides the participants and researchers. During the sessions, MYK followed the semistructured interview questionnaire covering topics related to the implementation of the MDSS (Textbox 1). The researchers followed interview guidelines based on previous research and approved by members of the eHealth research team at SNUBH.

### Data Analysis

**Figure 4:**
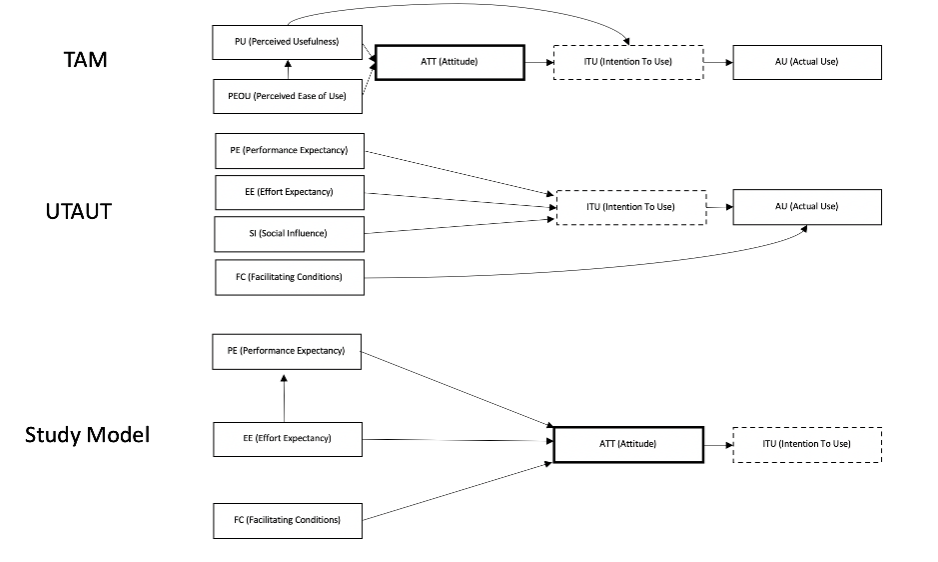
The analytical model used in this study, modified from the technology acceptance model (TAM) and the unified theory of acceptance and use of technology (UTAUT).

For SEM, the survey adopted the technology acceptance model (TAM) and the unified theory of acceptance and use of technology (UTAUT), both of which have been widely adopted to analyze user willingness to accept new technologies [15,29-32]. The models were modified to create a structural equation model optimized for this study Figure 4. Performance expectancy, effort expectancy, and facilitating conditions were expected to have a positive influence on attitude, and attitude was expected to have a positive influence on intention to use. The TAM includes two variables impacting behavioral intentions to use, and the UTAUT includes three behavioral variables and one variable that influences actual use, all of which influence the overall process. Theoretically, facilitating conditions should influence actual use. However, based on previous studies, we hypothesized that facilitating conditions would instead moderate intention to use due to difficulties in measuring actual use. Social influence from senior colleagues was omitted in order to simplify the model, as the study’s focus was on factors related only to user expectations and support from the hospital.

### Ethics

The research protocol was approved by the Institutional Review Board of Human Research of Seoul National University Bundang Hospital (Protocol No. B-1709-420-303).

## Results

### Participant Demographics

Table 2 presents the demographic characteristics of all SEM survey respondents. Of the 80 professionals invited to take the survey, 61 responded. Most were residents who use the EMR and MDSS more actively than any other position on the hospital staff.

For qualitative analysis, 15 people out of 80 participants were interviewed. Table 3 presents the interviewees’ demographic characteristics.

A reliability test was performed to confirm the consistency of the survey items for SEM analysis. Cronbach α exceeded .8 for all variables except facilitating conditions. Thus, the survey items were confirmed to be consistent and reliable (Table 4).

**Table 2:** Demographic characteristics of SEM survey respondents.

Categories/items		Number	Percentage
**Gender**			
	Male	22	36.07
	Female	39	63.93
**Age (years)**			
	20-29	13	21.31
	30-39	45	73.77
	40-49	2	3.28
	50 and above	1	1.64
**Department**			
	Internal/family medicine	42	68.85
	Pediatrics	8	13.11
	Surgery	5	8.20
	Other	6	9.84
**Length of service (years)**			
	<1	24	39.34
	1-3	30	49.18
	3-5	4	6.56
	5-10	0	0
	>10	3	4.92
**Position**			
	Professor	2	3.28
	Fellow	4	6.56
	Resident	55	90.16

**Table 3:** Demographic characteristics of focused interview participants.

Categories/items		Number	Percentage
**Gender**			
	Male	11	73
	Female	4	27
**Age (years)**			
	20-29	2	7
	30-39	12	80
	40-49	1	13
**Department**			
	Internal/family medicine	13	86
	Pediatrics	1	7
	Surgery	1	7
**Length of service**			
	<1	1	7
	1-3	12	79
	3-5	1	7
	5-10	1	7
**Position**			
	Professor	3	20
	Resident	12	80

**Table 4:** Reliability analysis.

Construct	Number of items	Cronbach α
Performance expectancy	3	.82
Effort expectancy	3	.90
Attitude	2	.86
Facilitating conditions	3	.70
Intention to use	3	.95

### Quantitative Analysis

Table 5 shows the total number of red and orange alerts presented by the MDSS each month from April 2017 to March 2018. A total of 185,441 red alerts (65.82%) were overridden.

Table 6 presents the usability test results. The overall mean score was 3.38. Study participants generally agreed with all statements except “I feel confident using Medi-Span,” which resulted in a positive score (above 3) but was not statistically significant.

**Figure 5:**
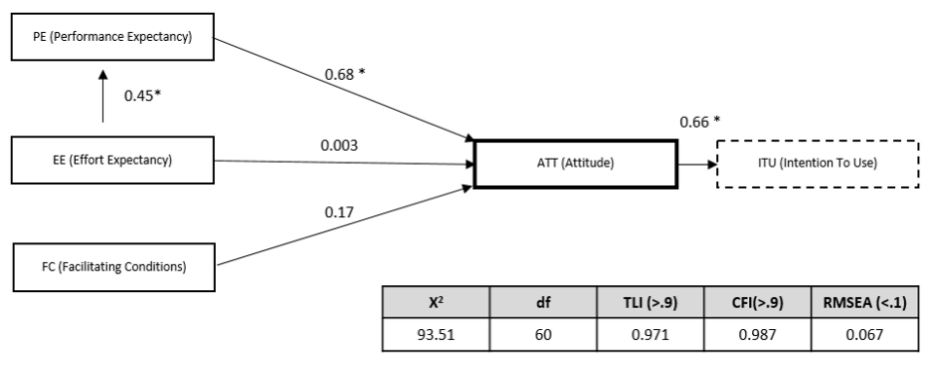
Results of the SEM model. *P<.001.

Figure 5 presents the overall results of the SEM analysis. For the research model, χ^2^ was 93.51 (*df*=60, *P*<.01), the Tucker-Lewis index (TLI) was 0.971, the comparative fit index (CFI) was 0.987, and the root mean square error of approximation (RMSEA) was 0.067. Because TLI and CFI exceeded 0.9, RMSEA was below 0.1, and the *P* value of the model was statistically significant, the model was confirmed to be appropriate for analyzing end-user intentions to use the MDSS. The associations between latent variables were positive and statistically significant, confirming the influence of performance expectancy on attitude, effort expectancy on performance expectancy, and attitude on the intention to use.

**Table 5:** Number of red and orange alerts presented by the MDSS each month.

Month	Red alerts (*N*)	Orange alerts (*N*)
April 2017	789	3805
May 2017	3979	29,037
June 2017	25,903	94,023
July 2017	23,868	73,986
August 2017	27,468	74,070
September 2017	24,401	69,403
October 2017	22,729	62,991
November 2017	25,536	72,996
December 2017	28,445	76,197
January 2018	27,728	82,671
February 2018	37,624	68,926
March 2018	3250	78,162
Total	1,126,724	281,720

**Table 6:** Usability test results.

Items^a^	Mean (95% CI)
I feel like I use Medi-Span frequently.	3.43 (3.23, 3.62)
Medi-Span is unnecessarily complicated to use.^a^	2.74 (3.08, 3.45)
Medi-Span is easy to use.	3.46 (3.31, 3.61)
I need technical support to use Medi-Span.^a^	2.70 (2.47, 2.93)
Medi-Span integrates various functions well.	3.41 (3.26, 3.56)
Medi-Span is not consistent in terms of usability.^a^	2.48 (2.31, 2.63)
I think most people learn how to use Medi-Span quickly.	3.51 (3.34, 3.68)
It is bothersome to use Medi-Span.^a^	2.59 (2.31, 2.67)
I feel confident using Medi-Span.	2.93 (2.78, 3.08)
It takes a long time to get used to Medi-Span.^b^	2.61 (2.45, 2.77)
Total score	3.38 (3.27, 3.48)

^a^Each item was rated on a 5-point Likert scale, with a score of 3 or higher indicating agreement with the statement (for questions with negative wording, a score of 3 or below indicated a positive response).

^b^Questions with negative wording were reverse-scored to calculate the mean total score.

### Qualitative Analysis

#### Alert Fatigue

Alert fatigue is a well-known problem associated with MDSS [20]. Participants in this study also mentioned alert fatigue several times.

It is inconvenient because there are many alerts for drugs commonly used in hematology-oncology, and it is not possible to dismiss the extreme caution alert, which frequently appears in older patients.

#### Lack of Accuracy

Accuracy is an essential factor affecting users’ trust in an MDSS. If false alerts pop up repeatedly, users will tire of the interruption and may fail to take heed when a valid alert is given. Accuracy is also closely related to alert fatigue because poor accuracy results in a higher number of unnecessary alerts. This study’s participants also mentioned accuracy frequently.

For example, when co-prescribing morphine and clopidogrel, the same message about drug-drug interaction occurs several times, and the alert override has to be selected several times.

#### Poor User Interface Design

South Korean medical staff are accustomed to using English at work. However, poor user interface design can display too much English information on one screen, making it difficult for professionals to see every message in a busy hospital setting. Particularly in emergent situations, poor user interface design presenting excessive and unnecessary English information can be problematic. Therefore, it is crucial to design a user interface that provides essential messages only. In this study, two participants mentioned that the context of English information was difficult to understand quickly.

It’s hard to understand the alert messages because they’re in English and include a lot of content.

#### Lack of Customizability

Participants highlighted the need for functionality in the MDSS to customize types of alerts according to user preference. To reduce the rate of overrides and alert fatigue, an MDSS must be easily customizable.

It would be nice to have the ability to set specific drugs and specific doses as a basis for alerts for each department or doctor.

## Discussion

### Principal Findings

This study employed a mixed-methods approach to analyze barriers and facilitators to the implementation of MDSS. Barriers were identified based on the results of SEM and qualitative analysis, and facilitators were identified based on SEM.

The quantitative analysis found an average usability rating of 3.38 out of 5, indicating acceptable usability of the MDSS. SEM analysis revealed that effort expectancy had a positive effect on performance expectancy, performance expectancy had a positive effect on attitude, and attitude had a positive effect on the intention to use. Thus, user expectations regarding ease of use may not directly affect their attitude. If users can utilize the system easily, they expect it to result in performance improvement, which in turn affects their attitude toward and intentions to use the system.

The qualitative analysis identified four significant barriers to implementation of an MDSS: alert fatigue, lack of accuracy, poor user interface design, and lack of customizability. To our knowledge, this is the first study to analyze barriers, facilitators, and usability of MDSS implementation based on a mixed-methods approach.

### Barriers

#### Alert Fatigue

A previous study revealed that medication safety alert fatigue could be reduced through interaction design and clinical role tailoring [33]. The results of our SEM analysis revealed that effort expectancy (ie, user expectations regarding ease of use) affects performance expectancy, which in turn affects intentions to use the system. If the problem of frequent alert fatigue is neglected, the usability of the MDSS will suffer, which will affect user performance expectancy and foster a negative attitude toward the use of the MDSS. In particular, previous studies have shown that the busy working environment of interns or residents can aggravate the adverse effects of alert fatigue [22].

#### Poor User Interface Design

Users clearly want to improve their work performance by using the MDS system. If they can utilize the MDSS to its fullest extent, they can expect to increase their work efficiency and performance. User interface design and experience are crucial to facilitate full utilization. If the system is difficult to use (ie, navigation is unintuitive), doctors will tend to dismiss or ignore messages from the MDSS. Previous studies have shown that user interface design is an essential factor in the successful implementation of a CDSS [34-36]. BESTCare has an integrated interface design, allowing users to easily and intuitively predict the next necessary action. The same interface design was adopted and upgraded to implement a third-party MDSS, helping users to quickly grasp the information presented by MDSS and move on to the next required action. However, the qualitative analysis found that redundant information hindered the user interface of the MDSS. Thus, the volume and layout of the displayed information must be considered in addition to the screen design.

#### Lack of Accuracy

A previous study found that 52.6% of MDSS alerts in outpatient clinics were overridden, 53% of which were appropriate [21]. Another study showed that 73.3% of patient allergy, duplicate drug, and drug interaction alerts were overridden in an inpatient clinic, only about 60% of which were appropriate [20]. Even if only 40% of overrides are considered inappropriate, this can significantly increase the risk of medication errors and potentially leading to ADEs. The override rate was 65.82% in the MDSS evaluated in this study, similar to previous results. Accuracy is related to performance expectancy. If accuracy remains consistently low, users will begin to lose hope of improving performance, resulting in a negative cycle of higher alert overrides.

#### Poor Customizability

BESTCare, the EHR integrated with the Medi-Span based MDSS used in this study, has an alert-related authority control function that meets the standards of the EHR certification program run by the Office of the National Coordinator for Health Information Technology. The professionals who participated in this study’s in-depth interview were dissatisfied with this integrated management system and wanted the ability to customize and adjust the alerts they received. MDS systems are usually introduced to prevent ADEs. Therefore, a centralized, integrated management system is necessary for consistency and stability. However, end-user satisfaction will increase if they can adjust the level of alerts provided without sacrificing this overall stability.

### Facilitators

#### Effort Expectancy

This study’s quantitative analysis found that doctors generally considered the MDSS easy to use. Before development, both hospital staff and developers expressed concern about integrating the new Medi-Span MDSS on top of the two MDSS already integrated into the EHR system because the integration of three MDSS within the EHR could result in an excessive number of alerts. However, dedicated trial and error by the taskforce team ensured the usability of the system. As revealed in the in-depth interview, the users’ wishes regarding MDSS usability can never be fully satisfied. However, the taskforce team’s activities to improve usability acted as important facilitators for the successful introduction of the MDSS.

#### Performance Expectancy

According to this study’s SEM analysis, end-user effort expectancy had a positive effect on their expectations of performance improvement. MDSS platforms must provide users with feedback on their actions in response to alerts and performance improvement outcomes in order to reduce overrides for valid alerts. Previous studies have noted that gaining user trust is crucial for the proper implementation and maintenance of a new system [37-39]. Clinical indicators regarding performance improvement and regular result reporting may be an excellent way to promote the use of the MDSS and gain trust. For example, public disclosure of antibiotic use rates effectively lowered the use of antibiotics for upper respiratory infections in South Korea [40]. Likewise, public disclosure about ADEs prevented by using the MDSS may help reduce alert override rates. Another option is to create a method by which users can provide feedback on false alerts. If doctors can provide feedback about false alerts instead of using the MDSS passively, the system can be dynamically upgraded to gain trust.

#### Limitations and Future Research

This study’s main limitation is that the system was implemented in only one hospital. External validation in other hospitals is needed to help generalize the study results. Nevertheless, this research demonstrated the effects of interaction between user expectations regarding ease of use and performance improvement on their attitude toward using an MDSS, which can inform the practices of system designers and policymakers in charge of MDSS development.

### Conclusion

This study revealed barriers and facilitators to the implementation of MDSS. The study’s findings can be used as a reference to upgrade MDSSs effectively. Further studies are needed to evaluate specific ways to gain MDSS users’ trust.

## Abbreviations

ADE: adverse drug event
API: application programming interface
ATT: attitude
AU: actual use
CFI: comparative fit index
COREQ: Consolidated Criteria for Reporting Qualitative Research Guidelines
EE: effort expectancy
EHR: electronic health record
EMR: electronic medical record
FC: facilitating conditions
GPI: generic product identifier
HIS: hospital information system
ITU: intention to use
MDSS: medication decision support system
PE: performance expectancy
PEOU: perceived ease of use
PU: perceived usefulness
RMSEA: root mean square error of approximation
SEM: structural equation modeling
SI: social influence
SNUBH: Seoul National University Bundang Hospital
TAM: technology acceptance model
TLI: Tucker-Lewis index
UTAUT: unified theory of acceptance and use of technology
